# Biomedical Applications of Quaternized Chitosan

**DOI:** 10.3390/polym13152514

**Published:** 2021-07-30

**Authors:** Kamla Pathak, Shashi Kiran Misra, Aayush Sehgal, Sukhbir Singh, Simona Bungau, Agnieszka Najda, Robert Gruszecki, Tapan Behl

**Affiliations:** 1 Faculty of Pharmacy, Uttar Pradesh University of Medical Sciences, Etawah 206130, India; kamlapathak5@gmail.com; 2 University Institute of Pharmacy, Chhatrapati Sahuji Maharaj University, Kanpur 208026, India; shashisarthak@gmail.com; 3 Chitkara College of Pharmacy, Chitkara University, Rajpura 140401, India; aayushsehgal00@gmail.com (A.S.); sukhbir.singh@chitkara.edu.in (S.S.); 4 Department of Pharmacy, Faculty of Medicine and Pharmacy, University of Oradea, 410028 Oradea, Romania; sbungau@uoradea.ro; 5 Doctoral School of Biological and Biomedical Sciences, University of Oradea, 410073 Oradea, Romania; 6 Department of Vegetable Crops and Medicinal Plants, University of Life Sciences in Lublin, 20-950 Lublin, Poland; robert.gruszecki@up.lublin.pl

**Keywords:** quaternized chitosan, antimicrobial, biocompatibility, biomedical, vaccine, wound healing

## Abstract

The natural polymer chitosan is the second most abundant biopolymer on earth after chitin and has been extensively explored for preparation of versatile drug delivery systems. The presence of two distinct reactive functional groups (an amino group at C2, and a primary and secondary hydroxyl group at C3 and C6) of chitosan are involved in the transformation of expedient derivatives such as acylated, alkylated, carboxylated, quaternized and esterified chitosan. Amongst these, quaternized chitosan is preferred in pharmaceutical industries owing to its prominent features including superior water solubility, augmented antimicrobial actions, modified wound healing, pH-sensitive targeting, biocompatibility, and biodegradability. It has been explored in a large realm of pharmaceuticals, cosmeceuticals, and the biomedical arena. Immense classy drug delivery systems containing quaternized chitosan have been intended for tissue engineering, wound healing, gene, and vaccine delivery. This review article outlines synthetic techniques, basic characteristics, inherent properties, biomedical applications, and ubiquitous challenges associated to quaternized chitosan.

## 1. Introduction

**Figure 1:**
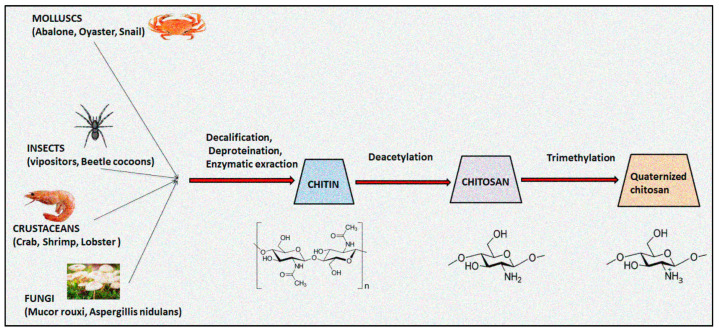
Schematic developmental process of quaternized chitosan from native chitin.

Chitosan, an aminoglucopyran polysaccharide, is widely utilized in pharmaceuticals, cosmeceuticals, biomedicals, agricultures, foods and packaging sectors owing to its inherent properties including biodegradability, biocompatibility, and non-toxicity [1,2]. The natural biopolymer chitosan is predominantly obtained from the exoskeletons of marine crustaceans, mollusks, insects, and fungi through an alkali deacetylation process. Figure 1 illustrated the schematic developmental process of quaternized chitosan from native chitin [3]. In contrast to natural occurring chitin, chitosan is very soluble in acidic solvents and fluroalcohols. Chitosan is a weak base, possessing pKa ranging from 6.2 to 7.0, and tends to be biodegradable in both in vitro and in vivo into non-toxic metabolites. It can be easily digested by lysozyme into non-active oligosaccharide and is thus a desirable component for designing absorbable sutures, osteoconductive implants and tissue scaffolds [4]. The biological functions of chitosan biopolymer are based on its molecular weight, degree of acetylation, charge density and extent of quaternization [5]. The physicochemical and biological properties of chitosan are primarily affected by the degree of deacetylation that directly impact on its molecular weight, pKa, crystallinity, hydrophilicity, degradation, and biological actions [6,7]. A degree of deacetylation value close to 0% or 100% prolongs biodegradation and cell adhesion, whereas transitional values of the degree of deacetylation display speedy degradation rates of chitosan. The physicochemical properties including the degree of acetylation and the molecular weight of chitosan and its derivatives are accountable for its biological responses [8]. USFDA-approved chitosan is highly utilized in tissue engineering, skin regeneration and wound healing drug delivery systems [9]. The presence of inter- and intramolecular hydrogen bonds demonstrates crystalline behaviour, which accounts for its poor aqueous solubility. The limited solubility in a wide range of physiological solutions restricts the use of chitosan in designing drug delivery systems [10].

Conformational changes in the chitosan skeleton are reliant on local environmental conditions including the pH, pKa, N-substitution group, process temperature and types of acids. The functional groups (hydroxyl and amino) contribute an essential role for imparting solubility to the chitosan molecule. At low pH, the attached amino group undergoes protonation, solubilizes and provides positive charge to the medium, offering strong electrostatic interaction with negative charge cell components. Moreover, the pKa of amino group highly depends on the degree of acetylation (DA); hence, the solubility of chitosan is also reliant on DA [11].

**Figure 2:**
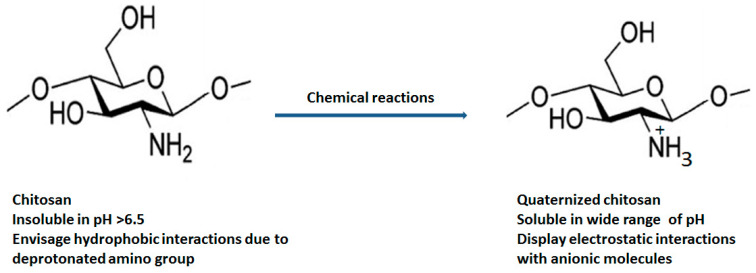
Structural differentiation between chitosan and quaternized chitosan.

Various chemical modification such as acetylation (insertion of anhydride and acyl chloride) [12], alkylation (alkyl group) [13], carboxylation (glyoxylic acid, chloroalkanoic acid) [14], quaternization (quaternary ammonium salts) [15], esterification (sulphuric acid, phosphoric acid and chlorosulfonic acid) [16] and etherification (chloroacetic acid, ethylene oxide, dimethyl sulphate) [17] at the C2, C3 and C6 position of chitosan are carried out to improve its physicochemical (enhanced aqueous solubility, improved absorption, high bioavailability, etc.) and biological properties (improved antimicrobial action and antioxidant, high penetration across cell membrane, mucoadhesiveness, etc.) Figure 2 displays the structural differentiation between chitosan and quaternized chitosan [18].

## 2. Quaternized Chitosan Derivatives and Physicochemical Properties

**Figure 3:**
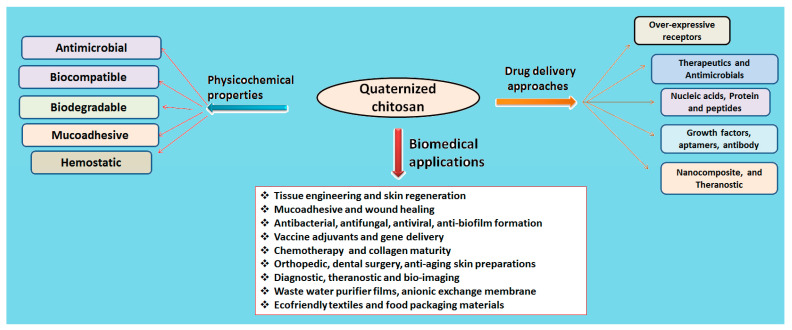
Quaternized chitosan and its physicochemical and drug delivery approaches.

The quaternization of chitosan involves the insertion of a hydrophilic group via any of three methods: direct quaternary ammonium substitution, epoxy derivative open loop and N-alkylation. A high degree of substitution provides better aqueous solubility and enhanced antimicrobial action, and lessens cytotoxicity with innate mucoadhesiveness and efficient penetration. The degree of quaternization and molecular weight are a few essential parameters that elicit physicochemical and biological actions (Table 1). Figure 3 compiles drug delivery approaches and biomedical applications of quaternized chitosan [19].

Quaternization not only enhances solubility but also escalates chargeability and antibacterial efficacy [22].

**Figure 4:**
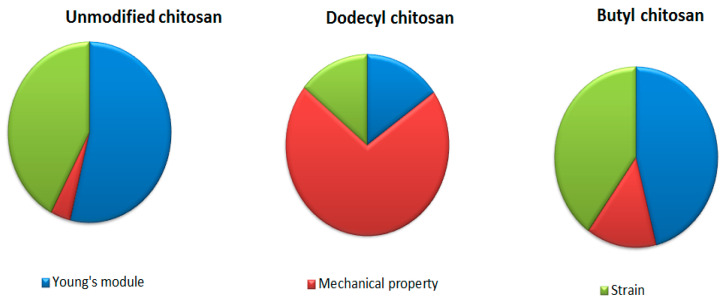
Comparative mechanical properties of chitosan and its derivatives.

The mechanical property plays a vital role in imparting biomedical application. It is observed that mechanical property of chitosan enhances on increasing its concentration owing to high crystallinity. Jana et al. (2012) investigated the comparative tensile strength of scaffolds fabricated from chitosan solution (4–12%). As the concentration of chitosan increased in scaffold, augmented mechanical strength (from 1.74 MPa to 17.99 Mpa) was displayed through XRD patterns. The degree of protonation and extent of crystallinity mediated the high strength of chitosan [23,24,25]. Britto et al. (2007) synthesized quaternary salts of chitosan through dimethylsulphate reaction. The mechanical strength, Young’s modulus and maximum strain were compared to the unmodified, N-alkylated and quaternized chitosan films, which are well depicted in Figure 4. Unmodified chitosan film exhibited higher mechanical strength compared to quaternized (N-dodecyl chitosan) and alkylated (butyl chitosan) in order of 44.0 Mpa > 38.3 Mpa > 13.4 Mpa, respectively [26,27,28].

### 2.1. N,N,N-Trimethyl Chitosan Chloride

The quaternization (trimethylation and triethylation) of decorated amino groups in molecular structure of chitosan is carried out by treating it with methyl iodide at a high temperature in alkali medium. Subsequently, the degree of quaternization of different grades of deacetylated chitosans is optimized through the modification of a number of reaction steps and time. N,N,N-trimethyl chitosan chloride (TMC), the most abundantly explored QCh derivative, has been utilized in the formulation of pharmaceutical products. It is obtained through the methylation of the amino group at the C2 site of chitosan backbone (Figure 4). QCH substituted through N-(3-chloro-2-hydroxy-propyl) trimethylammonium chloride has increased pharmacokinetic and antimicrobial properties. The extent of N-substitution (ES) affected antimicrobial action of QCH. The literature envisaged that when ES is 20% high, the efficacy of QCH derivative against pathogenic *E. coli* (16 to 64 μg/mL) and *S. aureus* (8 to 64 μg/mL) is enhanced. Furthermore, N-methylated, N-arylated, N,N-dimethylaminophenyl and pyridyl-substituted QCH are very soluble in water irrespective of the pH and exhibited superior antibacterial efficacy [29].

**Figure 5:**
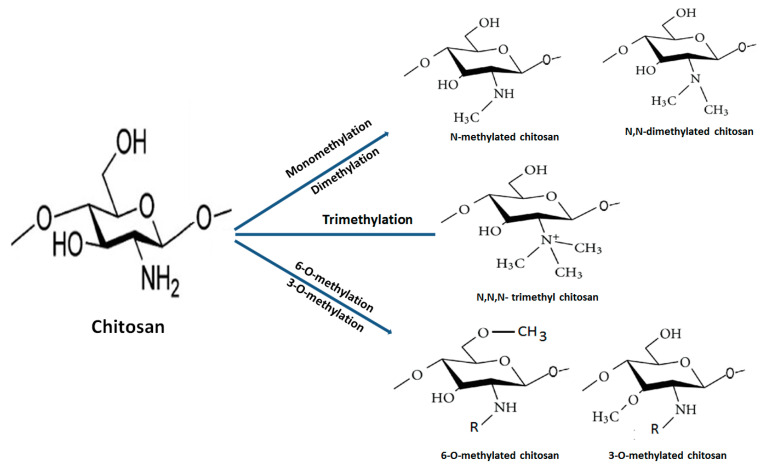
Different pathways for synthesis of methylated chitosan from native chitosan.

The multifunctional quaternized chitosan derivatives, their synthesis and biomedical applications are displayed in Figure 5 [30]. Other byproducts such as 6-O-methylated, 3-O-methylated and N, N-dimethylated chitosan are infrequently used due to their unfavourable properties. The very first TMC was synthesized by Terayama and coworkers and was called macramin [31,32]. A higher degree of quaternization achieved after O-methylation of chitosan at position 3-OH and 6-OH results in less soluble trimethyl chitosan iodide. The methylation process is considerably decreased through recycling of the basic reaction until the desired product is achieved. Furthermore, preparation of trimethyl chitosan (N,N,N-trimethyl chitosan) is accomplished through an ion exchange process that possesses high solubility and basicity. Reports have cited that the intermediate degree of quaternization (~40%) evidences the highest solubility regardless of the molecular weight and degree of acetylation of chitosan due to the replacement of the attached primary amino functional group with quaternary amino groups at the C2 position [33]. The molecular weight of TMC increases during the course of reductive methylation because the methyl group is added to the previously attached ammonium group of the repeated monomers. Moreover, a significant decrease in molecular weight is noticed after treating with strong alkali at an elevated temperature, which causes degradation of the polymeric chain. Furthermore, an increase in the degree of quaternization of TMC polymers causes a decrease in the intrinsic viscosity [34]. The mucoadhesive property decreases with an increase in the degree of quaternization, owing to the alteration of conformation of polymers during the quaternization process, which affects the interaction between fixed positive charges on each polymeric chain at C2 position. It also decreases the polymer–polymer flexibility. Furthermore, attached functional groups (methyl, ethyl, propyl, benzyl, etc.) on amino groups may hide positive charges and this causes a steric effect. Both decrease the polymeric chain flexibility and produce steric effects that influence the extent of charge transfer between cationic TMC and anionic sialic acid present in the mucus, thus resulting in inferior mucoadhesiveness [35].

### 2.2. N-[(2-Hydroxyl-3-Trimethyl Ammonium) Propyl] Chitosan

**Figure 6:**
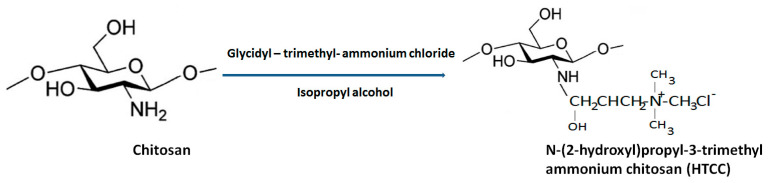
General synthesis of HTCC from biopolymer chitosan.

N-[(2-hydroxyl-3-trimethyl ammonium) propyl] chitosan or HTCC is the second most-popular QCh prepared via alkylation process involving insertion of quaternary ammonium group at the outside the chitosan structure. Lang et al. 1980 synthesized the first HTCC through chitosan modification [36]. The reagent ‘glycidyl trimethylammonium chloride’ (GTMAC) is often used for direct replacement of hydrogen from attached amino and hydroxyl functional groups from chitosan at the C6 position to obtain O, N-substituted quaternized chitosan (HTCC), which enhances the aqueous solubility and superior antibacterial efficacy (Figure 6) [37]. The generated HTCC polymers have different degrees of quaternization that refer to the extent of positive charge [38].

HTCC presents non-toxicity, improved aqueous solubility, and modified antimicrobial action along with biocompatibility and bioavailability features. Xiao et al. (2012) demonstrated that HTCC is more amorphous than crystalline chitosan. Quaternization weakens inter-/intra molecular hydrogen bonds and results in unorganized HTCC polymer that not only facilitates solubilization but also favours better absorption due to the diffusion of free water molecules from the HTCC chains [39].

Similarly, increased antimicrobial action is also defined due to abundant positive charge on HTCC that is attracted by negative surface charge present on the bacterial cell surface. HTCC encompasses permanent positive charges on its surface. The physicochemical properties of HTCC are highly reliant on the degree of quaternization. Wang et al. (2018) studied the effect of quaternization on the physicochemical and biological properties of HTCC. The outcomes revealed that the apparent viscosity, solubility, and elasticity are amplified by an increase in the degree of quaternization on storage. A lesser degree of quaternization produces hydrophobic HTCC polymer that is employed as an efficient vaccine adjuvant to adsorb antigens via hydrogen bonding formed through unoccupied amino groups [40]. Furthermore, Shagdarova et al. (2019) explained that a high degree of quaternization resulted in improved antibacterial action against *E. coli* and *S. epidermis* [41]. The versatile HTCC is widely combined with other chemicals such as poly (N-isopropylacrylamide) carboxylic acid, poly (l-caprolactone) and polyacrylonitrile to improve the solubility, tensile strength, and antimicrobial action for commercial purposes.

### 2.3. Pyridine Salt Grafted Quaternized Chitosan

Different new QCh-based pyridine salts have been derived that explore synergistic antiviral, antifungal and antibacterial action owing to the attached cationic pyridine salts. In this context, PACS (pyridine chloroacetyl chitosan), BHPACS (4-(5-bromo-2-hydroxybenzylideneamino)-pyridine) and CHPACS (4-(5-chloro-2-hydroxybenzylideneamino)-pyridine) synthetic quaternized chitosan derivatives have been derived from reacting chloroacetyl chitosan and pyridine. These synthetic QCh-based substances exhibit strong antifungal potency towards *Monilinia fructicola*, *F. oxysporum*, *Cladosporium cucumerinum* and *Colletotrichum legenarium* [42]. The antifungal effect occurred through polycationic-charged pyridine consisting of QCh and negatively charged fungal cell surface. The sturdy electrostatic interaction causes the disorganization and denaturation of proteins present in cell membranes, and ultimately cell death. The literature also reports the synthesis of N-methyl-pyridinium and N-methyl-1, 2, 3-triazolium grafted QCh derivatives that were quite effective for phytopathogenic fungi including *F. wilt*, *Collectotrichum lagenarium* and *F. oxysporum*.

**Figure 7:**
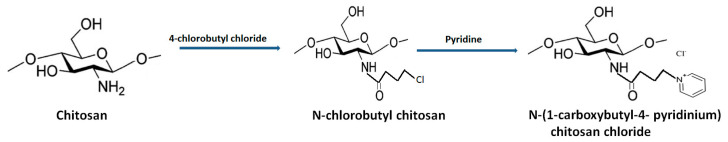
Synthetic route for the development of pyridine-grafted quaternized chitosan.

The presence of a pyridine ring and 1, 2, 3-trizolium in the QCh backbone depicted enhanced solubility in alkaline medium and mediated superior antifungal action compared to unmodified chitosan [43]. Jia et al. (2016) synthesized N-(1- carboxbutyl-4-pyridinium) chloride chitosan through introducing nucleophilic substitution on pyridine molecules (Figure 7 [44]. The antifungal activity against *B. cinerea* and *F. fulva* revealed disturbance in cell permeability owing to the adsorbed cationic charges on the fungal cell surface. Consequently, deformation of fungal hypha and cell death was reported [45].

### 2.4. Phosphonium Salt Grafted Quaternized Chitosan

**Figure 8:**
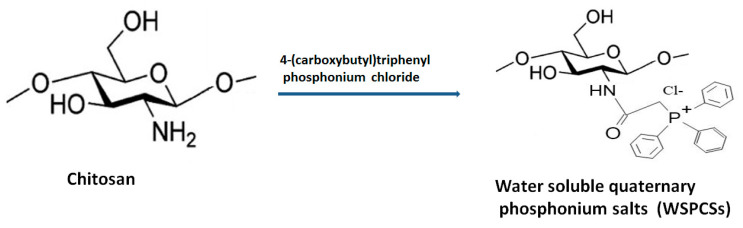
Synthesis of WSPCS from chitosan molecule.

Multifunctional quaternary phosphonium salts are organophosphate biocides, basically obtained from the nucleophilic substitution reaction between halide salts and phosphine. These compounds have better antimicrobial action than QCh polymers and are employed in gene therapy, vaccine adjuvant and in water purification units. One of the reported water-soluble quaternary phosphonium salt (WSPCS) has been synthesized from QCh with a minimal degree of quaternization (~4%). The developed WSPCS also exhibited better solubility in organic solvents. Figure 8 illustrates the basic pathway for the synthesis of WSPCS. Increasing the substitution resulted in low cytotoxicity (for mouse fibroblast) and greater antimicrobial action [46]. The cross-linked quaternary-phosphonium chitosan is widely used in wastewater treatment as the phosphonium ion has greater efficiency for the adsorption of chromium ions. Zeng et al. developed quaternary chitosan phosphonium particles loaded with pEGFP (plasmid type enhanced green fluorescent protein) for the formulation of novel gene vectors. The synthesized particles exhibited biosafety and high potential for gene transfection [47].

### 2.5. Other Quaternized Chitosan Derivatives

N-betainates and mono/diquaternary piperazine derivatives of chitosan revealed a varied degree of quaternization without any side reactions. The development of these derivatives provided well-defined structural determinants that are accountable for extreme solubility (4–6% *w*/*v*) at pH = 7 and better absorption/permeation enhancer [48]. Table 2 enlists novel quaternized chitosan derivatives and their biomedical usage.

Rahimi et al. (2019) introduced QC-IMDZ, a novel quaternized chitosan, after grafting quaternary imidazole into the hydroxyl groups of chitosan backbone. Wound dressing material embedded with silver nanocomposite on the QC-IMDZ surface was fabricated that showed superior antimicrobial actions for both bacteria and fungi due to the presence of cationic imidazole. However, excess silver nanocomposite embedded film showed lower solubility and required a prolonged clotting time compared to a low amount silver-nanocomposites-embedded QC-IMDZ films [49].

**Figure 9:**
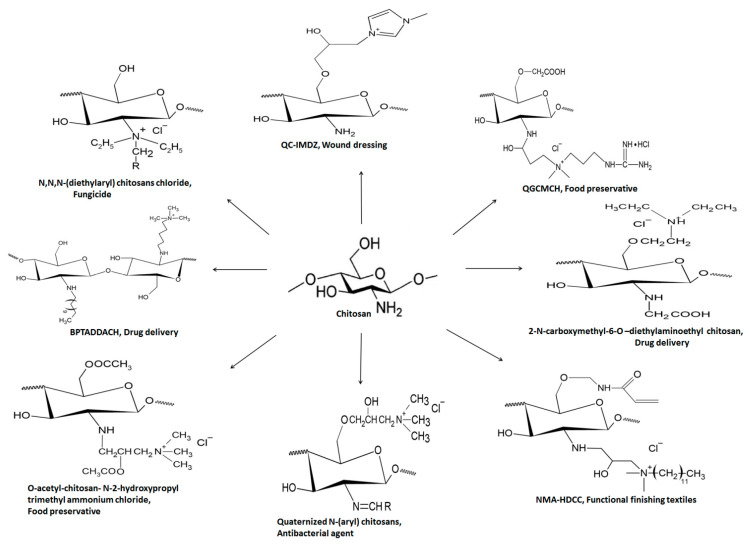
Structural illustration of different QCh derivatives and their biomedical applications.

Another quaternized chitosan derivative ‘N-O-[N,N-diethylammoniomethyl (diethyldimethylene ammonium) methyl chitosan has the efficiency to modify the penetration of drug candidates irrespective of their affinity towards mucosal epithelium. Improved drug penetration through porcine buccal epithelium was observed via the paracellular route [50]. Furthermore, ex vivo and in vivo studies suggested satisfactory permeation of dexamethasone in rabbit cornea through the transcellular transport route compared to widely used TMC [51]. Figure 9 summarizes structural and biomedical applications of diverse QCh derivatives synthesized from natural chitosan. Figure 9 compiles a few quaternized chitosan derivatives and their biomedical applications [52,53].

Wang et al. (2016) developed five aqueous soluble quaternized chitosan derivatives (O-quaternary ammonium salt-chitosan) that contained N-methyl-N-R-N-bis(2-hydroxyethyl) ammonium bromide. Grafted R substituents were benzyl chloride, dodecyl, tetradecyl, hexadecyl and octadecyl. All the O-quaternized derivatives exhibited greater solubility, better antibacterial action for Gram-positive bacteria and lower cytotoxicity against AT2 cell lines. Furthermore, dodecyl- and tetradecyl-grafted O-quaternary ammonium salt chitosan exhibited better biological properties compared to other derivatives and suggested a facile way to design QCh-based hydrogels, membranes, coated beads, scaffolds, dressing materials and bandages and nanoparticles owing to extreme solubility, permanent cationic charges, and characteristic antibacterial actions [67].

Few fatty acids were conjugated on quaternized chitosan nanoparticles for the efficient insulin delivery in liver. Strong electrostatic interaction between positively charged quaternized chitosan and negatively charged insulin was observed. Furthermore, the addition of fatty acids increased the surface hydrophobicity, which mediated hepatocyte absorption, enhanced insulin accumulation in liver cells and illustrated a better antidiabetic effect. Insulin-embedded quaternized chitosan nanoparticles consisting of lauric acid and oleic acid exhibited impressive bioavailability of 233% and 311%, respectively, after subcutaneous administration [68].

## 3. Biomedical Applications of Quaternized Chitosan Derivatives

### 3.1. Antimicrobial

The chemical configuration of polycationic QCh is a desirable requisite for the antimicrobial action. Electrostatic interaction between cationic QCh and anionic microorganisms plays an important part for antibacterial activity. A higher degree of substitution of ammonium groups adorned on the backbone of QCh imparts a positive charge that neutralizes cell surfaces of bacteria and disturbs cytoplasmic integrity [37,69]. Moreover, hydrophobic alkyl substitutions on QCh stimulate significant bacterial death as they preferably interact with the inner surface of the bacterial cell wall. The antibacterial effect is prominent in neutral and high pH rather than acidic. The literature envisages that high-molecular-weight and solid QCh derivatives are unable to pass through the cell membrane. They are only adsorbed at the microbial cell surface, channelize electrostatic interactions, hamper nutrient transport, and cause alteration in cell permeability. Conversely, low-molecular-weight water-soluble QCh particles penetrate the bacterial cell wall, intercalate with DNA, and inhibit the transcription process [70]. QCh derivatives always remain positively charged and are soluble at all physiological pH.

The antibacterial activity of the very first N,N,N-trimethyl chitosan was reliant on the degree of quaternization from the [−N(CH_3_)_3_] structure present in the molecule. A higher degree of quaternization improved its aqueous solubility. Additionally, the presence of hydrophobic methyl groups increased the interaction with the lipoidal cell membrane of the microorganism and depicted superior antimicrobial action [71]. Another derivative, N,N-di-ethyl-N-methyl quaternized chitosan (DMCHT) was synthesized via reductive alkylation of aldehydes, through the formation of Schiff base. The developed QCh exhibited antimicrobial efficacy owing to the formation of polyelectrolyte complexes between QCh derivative and peptidoglycan of bacterial cell, which, in turn, inhibited the growth of bacteria [72]. The antimicrobial efficacy of TMC and DMCHT was compared at 50% degree of quaternization. The results showed superior antibacterial activity of TMC against *S. aureus* owing to the presence of smaller alkyl groups that enabled easy interaction with the cell wall of the bacteria. Voluminous DMCHT was comprised of heavy N-ethyl functional groups on its structure [73].

Furthermore, comparative antimicrobial efficiency was assessed among DMCHT (N, N-di-ethyl-N-methyl quaternized chitosan), BZDCHT (N-benzyl-N-N-di methyl chitosan) and BDCHT (N-butyl-N,N-dimethylchitosan) against *S. aureus* and *E. coli* at pH 7.4. The outcomes displayed prominent antimicrobial activity of these QCh as compared to chitosan. DMCHT and BDCHT showed higher hydrophilicity required for better antimicrobial efficacy in comparison to BZCHT. Insertion of hydrophobic groups (phenyl and benzyl) decreased the antimicrobial action by virtue of shielding interaction between N-quaternized site and the cell wall of the bacteria [74]. Reports cite that the protonated amine groups including +NH_3_ and +ND/+NM (non-quaternized amine group) are highly effective antibacterials compared to +NT (N-trimethylated) ones. However, the chains of TMC derivatives are more flexible and readily interact with the bacterial cells than chitosan at pH ≥ 5.5 suggesting promising antibacterial potential where neutral pH required [75].

Fu et al. (2011) synthesized O, N-quaternized chitosan through attaching benzyl radical on N-quaternized chitosan followed by treatment with ethyl iodide in the presence of alkali at 36 °C. Developed cationic O,N-quaternized chitosan possessed enormous quaternary ammonium chloride moieties that interacted with negative residues of *S. aureus* cell surface. Therefore, affected cell integrity (protein, lipopolysaccharide and techoic acid), altered plasma membrane permeability, leakage of nutrients and mediated cell death [76]. Similar antibacterial action was observed for Gram-negative *E. coli* that contains a thick layer of extracellular lipopolysaccharide and checks the entry of foreign molecules. O-quaternized chitosan exhibited slightly less antibacterial action compared to O,N-quaternized chitosan derivatives by virtue of the reduced electron donation through N-benzyl modification [77,78].

Several natural and synthetic polymers are utilized either alone or in combination for the development of antibacterial systems [79]. In this context, the fabrication of non-woven nanofibers from QCh derivatives; i.e., N-(2-hydroxy)-propyl-3-trimethylammonium salt of chloride has been employed to elaborate wound healing properties of biomaterials including hydrophilicity, water retention capacity, mucoadhesiveness, antimicrobial efficacy, etc. [80]. However, the presence of ionogenic groups in quaternized chitosan generates high repulsive force that limits uniform fabrication of nanofibers via electrospinning. Thus, non-ionic polymers (poly-L-Lactide-co-d and poly vinyl alcohol) are incorporated to make the polymeric solution electrospinnable [81].

### 3.2. Antiproliferative

Biocompatible quaternized chitosan displays adequate antimicrobial and antiproliferative activity both in vitro and in vivo. For instance, QCh-coated sutures exhibited comparatively high anti-infection and cytocompatibility compared with triclosan-coated sutures, owing to the presence of ample positive charge on decorated quaternary ammonium functional groups that interact with the negatively charged phosphoryl groups of microorganisms [82]. Triclosan, a widely used antibacterial agent, has a significant advantage over other antibiotics by virtue of its low drug resistance and potent inhibition of biofilm formation. However, triclosan-coated sutures are effective in controlling surgical site infections, but the occurrence of tissue toxicity and induction of tumor proliferation and endocrine disorders limit its practice in surgical operation. The above investigation favored the usage of broad spectrum antibacterial QCh as an alternative to triclosan in orthopedic surgery [83].

Yang et al. (2016) elucidated limitations associated with vicryl plus (triclosan coated polyglactin) sutures such as surgical site infections and toxicity. Vicryl plus sutures were modified through surface coating of QC. Bacterial cell adhesion efficiency of modified HV sutures against *Staphylococcus epidermidis* and *S. aureus* revealed significant antibacterial efficiency for both pathogens for 48 h. Furthermore, the HV sutures were found to be cytocompatible with human-skin-derived fibroblast cells. The procedural flexibility and economic value of QCh-modified HV sutures laid down a suitable strategy to manage suture-related infections in orthopedic surgeries [84,85].

Furthermore, Li et al. (2018) focused on the clinical application of QCh in malignant melanoma (B-Raf^V600E^), one of the most invasive skin carcinomas. Human melanoma cells were interacted individually with vermurafenib (B-Raf^V600E^ inhibitor), QCh and a combination of both, to investigate cell permeability, proliferation, apoptosis, and viability. The outcomes revealed that the presence of QCh promoted an antiproliferative effect against melanoma cells. Moreover, QCh could enhance cell permeability at the preliminary stage, thus facilitating intracellular drug uptake. Interestingly, polycationic QCh made electrostatic interactions with negatively charged cancerous cells that disturbed their cytoplasmic integrity. The investigation concluded that QCh upset the surface charges on melanoma cell, which may be an important parameter to change cell permeability [86]. Wongwanakul et al. (2017) examined cell proliferation and cell differentiation properties of QCh on the intestinal barrier through CaCo-2 cell lines in vitro model. Poor cytotoxicity and superior biocompatibility on the intestinal barrier were observed from a lesser degree of substitution and low dose of QCh. The study suggested potential usage of QCH in designing oral drug delivery system [87].

### 3.3. Antibiofilm

Biofilms, the complex colonies of living microorganisms developed at the infection site, are extremely resistant to antibiotics and antimicrobials. These biofilms consist of a well-recognized self-produced extracellular matrix made up of protein, polysaccharide, and DNA [88]. Bacterial adherence and the biofilm formation at the implantation site involve two typical processes. Initially, the accumulation of microbial community starts, which releases polysaccharide intracellular adhesion, an extracellular substance. Furthermore, PIA is mediated through intracellular adhesion that comprised of different core genes such as ica A, B, C, ica D and one regulatory gene icaR. The icaA gene is an index of biofilm preparation. A high concentration of QCh is capable of preventing icaA transcription, thus limiting biofilm formation or microbial viability [89].

Biofilms produced at the post-surgical infection sites are mostly associated with the use of medical devices including catheters, implants, endotracheal tubes, valves, etc. Both Gram-positive (*S. aureus*, *S. epidermis*) and Gram-negative (*P. aeruginosa*) bacterial communities grow on healthcare devices [90]. These pathogenic bacteria can survive even in the presence of high doses of antibiotics (1000 times high) than required to eliminate their planktonic population. Once well-organized biofilm is formed around the surgical implants surface, it becomes difficult to completely rule out through antibiotic therapy [91].

Most of the existing antibiotics and antimicrobials are unable to eradicate biofilms, owing to their similar activity spectrum and common modes of action. QCh can be employed for the elimination of biofilm by virtue of its anti-infective property against bacteria, viruses, and fungi [77,92]. Both high- and low-molecular-weight QCh functionalized with hydrophobic residues (thiol protected 6-mercaptonicotinamide and methylated cyclodextrins) are reported as unconventional antimicrobial agents as they display improved wound healing, mucoadhesiveness and antibiofilm potential [93].

Piras et al. (2019) proved the biomedical application of QCh to be possible, i.e., antibacterial, anti-adhesive and antibiofilm. They demonstrated collective data of high- and low-molecular-weight QCh and their efficacy against common pathogens including *P. aeruginosa* and *S. epidermis*. Low-molecular-weight QCh derivatives were efficacious biofilm eliminators at very low concentrations, whereas high-molecular-weight QCh after covalent immobilization on titanium plates could be applied as spray or liquid plasters for the inhibition of bacterial adhesion. Thus, QCh-modified titanium implants mediated the dynamic strategy to terminate biofilm associated with medical devices [94].

### 3.4. Antifungal Activity

As with antibacterial activity, the occurrence of ample positive charge on QCh supposed to interact with the anionic residues of macromolecules present on the fungal surface resulted in the leakage of intracellular electrolytes and nutrients. Reports cite that QCh affects morphogenesis of the fungal cell wall and controls the functions of enzymes accountable for growth [95]. Guo et al. (2007) synthesized different QCh derivatives for the study of antifungal activity against *Botrytis cinerea* and *Colletotrichum lagenarium*. Four QCh derivatives, i.e., N-(2-hydroxyl-phenyl)-N,N-dimethyl CS, N-(5-bromic-2-hydroxyl-phenyl)-N,N-dimethyl CS, N-(2-hydroxyl-5-nitro-phenyl)N,N-dimethtyl CS and N-(5-chloro-2-hydroxy-phenyl)-N,N-dimethyl CS, were developed, which exhibited better antifungal effects compared to the unmodified chitosan [96]. The result emphasized that high molecularity of QCh is accountable for stronger antifungal efficacy [97].

Insertion of an arylfurfural group to the N-quaternized chitosan indicated amplified antifungal effects compared to unmodified chitosan. Moreover, N-quaternized arylfuran chitosan (QACHT) substituted with chlorine and nitrogen oxide further modified the antimicrobial and the antifungal effects that interacted with anionic macromolecules of fungal cell wall and mediating seepage of intracellular electrolytes [98]. Deacetylated chitosan functionalized with propyl and pentyl trimethylammonium bromide exhibited increased antifungal activity, i.e., three- and six-fold higher for *A. flavus*. In vitro antifungal assay evaluated the minimum inhibitory concentration for 72 h by varying the QCh derivatives concentration (0.5–16 g/L). The outcomes revealed that QCh derivatives inhibited mycelium growth even at one quarter of the concentration of deacetylated chitosan [99].

The antifungal activity of QCh can also be improved by the addition of more quaternary ammonium groups such as N-trimethyl. Increased molecular weight, positive charge, degree of quaternization and hydrophobic functional moieties intensify antifungal action. Il’ina et al. (2017) synthesized synthetic metal (Cu II) complexes of QCh (N-propyl chitosan derivative) and evaluated the antifungal efficacy against yeast (*C. albicans* and *R. rubra*) and mycelial fungi (*F. oxysporum* and *C. herbarum*). N-propyl-derived QCh was developed through treatment with glycidyl trimethyl ammonium chloride under controlled conditions. Amalgamation of N-propyl QCh (53%) and copper ions (13%) proved efficient against common fungal plant pathogen *F. oxysporum* [100]. Viegas de Souza et al. (2017) synthesized low-molecular-weight QCh derivative (dodecyl aldehyde-treated propyl trimethyl-ammonium bromide chitosan) to investigate the antifungal effect on *A. parasiticus* and *A. flavus*. The outcomes revealed that amphiphilic QCh derivatives exhibited amplified inhibition indices that were reliant on hydrophobicity and polymer concentrations. These QCh derivatives opened novel avenues for the development of chitosan-based biofungicides [101].

### 3.5. Mucoadhesiveness

N,N,N-trimethyl chitosan is widely utilized as a penetration enhancer for the delivery of peptides and macromolecular compounds across the mucosa in alkaline and neutral pH medium. Here, the degree of quaternization has a direct impact on the property of mucoadhesiveness and penetration across the membrane. Snyman et al. (2003) studied the effect of extent of quaternization (22–48%) and molecular mass (100,000 g/mole) on the biological activity of synthesized TMC polymers. The outcomes suggested decreased mucoadhesiveness on increased quaternization of TMC polymers [35,102].

The ocular or ophthalmic drug delivery system encounters several route barriers related to nasolacrimal drainage, stimulated lacrimation, blinking, blood ocular barrier and corneal impermeability. The use of mucoadhesive polymers such as chitosan derivatives facilitates effective ocular drug delivery owing to their elite interaction with the mucosal membrane. The presence of positive charge mediates the electrostatic interaction with the anionic mucin of the mucosal layer [103]. The ocular drug absorption of therapeutics depends on their aqueous solubility and mucoadhesiveness. Quaternized chitosan shows better candidature for enhanced permeation across ophthalmic tissues owing to their improved solubility and mucoadhesive property. However, beta-cyclodextrin conjugated chitosan has been employed for the ocular delivery of dexamethasone but restricted mucoadhesive limited their use.

Piras et al. (2018) demonstrated the development of methyl-beta-cyclodextrin grafted quaternary ammonium chitosan that was processed through treatment with hexamethylene diisocyanate. The QCh derivative efficiently carried dexamethasone, exhibited drastic changes in solubility and bioavailability. Moreover, a stable, mucoadhesive and controlled release formulation could be achieved. Preliminary assessments revealed that the developed conjugate has promising potential for the effective management of ocular disorders [93]. Yostawonkul et al. (2017) developed mucoadhesive oleoyl- quaternized chitosan-coated nanostructured lipid carriers (NLCs). Alpha-mangostin, a hydrophobic drug was embedded in the developed NLC, which exhibited more than 90% entrapment efficiency and minimum polydispersity. Excellent mucoadhesive, higher physical stability and greater cytotoxicity were observed against cancerous Caco-2 cells over Hela cells [104].

Konovalova et al. (2018) analyzed the comparative mucoadhesive property of chitosan (20 kDa) and its hydrophobic and quaternized hydrophobic chitosan derivatives (HC and QHC). The unmodified chitosan exhibited large aggregates in the keratinocytes and colon cells of murine small intestine, whereas HC and QHC showed mucoadhesiveness as a fine dotted line when observed through confocal microscopy [105]. Hydrophobic derivatives of chitosan mediated local drug release and improved biodegradation.

### 3.6. Drug Carriers

#### 3.6.1. Nanofibers

Nanofibers have always been a lucrative tool for the preparation of scaffolds, either as topically applied dressing material or in the membrane unit for filtration. Water purification systems enclosing membrane filtration units are widely accepted strategies for the retention of pathogens including bacteria and viruses according to their size. Table 3 compiles few novel biomedical usages of QCh-based nanofibers. Reports cite that holding small-sized viruses (<25 nm) necessitates enormous membrane surface area along with low water flux and high transmembrane pressure. Moreover, the filtration membrane unit has to be replaced with another new membrane frequently. To overcome this downside, Bai et al. (2013) fabricated an electro spun nanofibrous membrane comprised of quaternized chitosan polymer (HTCC) and graphene allotrope. HTCC has the proficiency to adsorb pathogenic non-enveloped porcine parvovirus on its surface. Distinctive attributes of graphene hydrophobicity and ionogenic HTCC enhanced the functional efficiency of developed nanofibers (95% virus retention). The developed nanofibers embedded with a blend of HTCC/graphene depicted effective microfiltration membrane water purification systems featured with low pressure technology for the significant removal of pathogens [106].

Santos et al. (2018) extended the study and fabricated antibacterial nanofibers from a blend of N-(2-hydroxy)-propyl-3-trimethylammonium chitosan chloride (QCh) and polycaprolactone (PCL) polymeric solutions. The produced nanofibers displayed improved hydrophilicity and tissue compatibility with superior mechanical strength when evaluated for enzymatic degradation, cell adhesion, and cytotoxicity against neonatal human dermal fibroblasts cells. The viscosity and degree of deacetylation of QCh strongly affected the alignment of nanofibers that was required for relative orientation with cancerous cells for better cytotoxic activity. The fabricated QCh/PCL nanofibers possessed essential benchmarks required for designing wound dressing materials in the domain of tissue engineering [107].

Amplified antimicrobial efficiency of quaternized chitosan was exploited by Cheah et al. (2019) as they performed surface grafting of PAN nanofibers (P-CN) with quarternary amine (glycidyl trimethyl ammonium chloride) functionalized chitosan under acidic, neutral, and alkaline conditions. Developed water-stable quaternized chitosan nanofibrous membranes (P-HTCC) were subjected for evaluation of antibacterial activity against *E. coli*. The outcomes from the microbiological assessment revealed that the quaternized chitosan-derived membrane developed in acidic medium exhibited stronger antibacterial action (99.95% for *E. coli*) compared to the neutral and alkaline medium. The fabricated P-HTCC membranes had the potential to be assembled into a microfiltration unit for the disinfection of Gram-negative pathogen *E. coli* [108].

Mi et al. (2014) addressed the issue of unsafe drinking water containing countless microbial loads and toxic impurities accountable for millions of deaths every year. An economical antiviral filtration material was fabricated utilizing ionogenic quaternized chitosan (HTCC) and non-ionogenic polyvinyl alcohol (PVA). The electrospun material embraced the high surface area required for effective water adsorption. Furthermore, HTCC/PVA nanofibers were treated with glutaraldehyde (cross linking agent) to control the water solubility and enhance the water stability (only 30% swelling in 6 h). The system could retain both enveloped and non-enveloped viruses. The HTCC-/PVA-embedded microfiltration membrane was affordable and proved to be an efficient low-pressure filtration unit by virtue of significant retention of pathogens [109].

#### 3.6.2. Hydrogel

Hydrogels have a cross-linked network of variable hydrophilic functional groups containing polymers that can absorb plentiful water. Surface-adorned hydrophilic groups such as amine (–NH_2_), hydroxyl (−OH), sulphate (−SO_3_H), and amide (−CONH−) empower hydrogel to absorb watery fluid that expands their volume due to swelling. Hydrogel-based dressing materials provide several advantages, including ample absorption of tissue exudate, maintenance of optimum moisture content at the site and encouraging cell proliferation (Table 4). Antibacterial hydrogels are highly required in the health care sector to accelerate the wound healing cure rate. In this context, Xiao et al. (2020) prepared hydrogel consisting of QCh, chemical cross-linker ‘polyacrylamide’, and silver nanoparticles. The developed hydrogel exhibited desirable tensile strength (approximately 100 kPa) with a shear stress of 10^4^ Pa. Excellent swelling capacity, a synergistic antibacterial effect, and low toxicity of Ag-mediated hydrogel reinforced the designing of appropriate wound dressing materials [115].

You et al. (2016) synthesized quaternized chitosan via reaction of chitosan with 3-chloro-2-hydroxypropyltrimethylammonium chloride in an alkaline solution. Thereafter, the polyelectrolyte complex hydrogel was prepared through in situ polymerization of poly acrylic acid monomers in quaternized chitosan solution. Resultant elastic, mechanically strong and surface tunable hydrogel was developed that displayed superb solvent-induced shape memory performance [116].

Mamidi et al. (2021) demonstrated a pH- and thermal-responsive chitosan-based nanocomposite (CNP) hydrogel that has potential applications in nanomedicine and nanotechnology. They developed an integrated chitosan nanocomposite containing poly (N(4-aminophenyl) methacrylamide)- carbon and diclofenac. The system possessed several conductive physicochemical properties such as spherical shape and uniform particle size. Additionally, the CNPs were pH- and heat-sensitive and exhibited controlled release for 15 days. Moreover, the designed CNP showed significant cell viability for human fibroblast cells [117].

#### 3.6.3. Beads

Thermoduric bacteria that can survive even after pasteurization of milk pose momentous threat to dairy and beverages industries. To overcome this challenge, biodegradable HTCC-anchored magnetic cellulose beads were developed via the dropping technique which could resist temperature up to 300 °C. Extended antibacterial efficacy against *Alicyclobacillus acidoterrestris* suggested the potential application of developed beads for food safety management [125]. Furthermore, quaternized chitosan beads were developed to adsorb phosphate and nitrate ions present in aqueous solution. Quaternized chitosan beads were formulated through reacting cross-linked chitosan with trimethyl ammonium chloride. The quaternized chitosan beads were quite effective in the pH range of 3–9 and exhibited an adsorption capacity for phosphate (97.5%) and nitrate ions (99%) that followed Freundlich isotherm model. The presence of common ions such as chloride, sulphate and bicarbonate did not alter the sorption capacity of quaternized chitosan beads [126].

The literature envisages that polycationic ammoniated polymers are fascinating options for the adsorption of negatively charged ions and sulphated polysaccharides through electrostatic interaction. In this regard, QCh has been explored for capturing anions, even at high pH. Eskandarloo et al. (2018) proposed the formulation of quaternized chitosan/polystyrene microbeads (CS/PS) for the selective adsorption of heparin, an anticoagulant from porcine intestinal mucosa sample. The comparative adsorption efficiency of CS/PS microbeads and marketed Amberlite FPA98 Cl resin was evaluated utilizing a heparin-bovine serum albumin model in pH range 4.1–9.2. The outcomes depicted superior adsorption efficiency of CS/PS micro beads (2.84 mg/g) and could be regenerated after treating with sodium chloride solution. Furthermore, the recovered microbeads can be reused for adsorption of heparin without any loss of adsorption capacity. Moreover, CS/PS microbeads could adsorb heparin from real biological sample containing heparin [127].

Quinlan et al. (2016) utilized quaternized chitosan hydrogel beads for efficient adsorption of 2-naphthoxyacetic acid from oil sand processed water (OSPW) system. 2-naphthoxyacetic acid is highly toxic and occurs in significant concentrations (40–70 mg/L) in OSPW that requires prolonged time for degradation. Hence, it necessitates removal of 2-naphthoxyacetic acid through either filtration, advanced oxidation, or adsorption methods. QCh has been identified as an attractive adsorbent by virtue of adsorption of anionic ions. The research emphasized retention of aromatic organic carboxylate site present in 2-naphthoxyacetic acid through polycationic QCh via the ion exchange adsorption process. The mode of adsorption signified Langmuir isotherm following pseudo first-order kinetics. The developed QCh hydrogel beads were moderately swellable and demonstrated effective adsorption of 2-naphthoxyacetic acid (91%) at the initial concentration of 200 mg/mL [128].

#### 3.6.4. Nanoparticles

Splendid accomplishments have been anticipated through chitosan-based nanoparticles for the management of different diseases over past decade. These biodegradable and biocompatible nanoparticles are not only exhibit improved solubility, site specific/localized action but also minimize undesirable toxicity. Highly demanded chitosan is frequently explored as a carrier in drug delivery systems, fabrication of wound dressing materials, management of skin regeneration and tissue engineering. Quaternized chitosan-based nanoparticles (QCh NPs) have attracted wide interest owing to their exclusive physicochemical and biological features. Chemical modification such as grafting, functionalization, Schiff base formation and quaternization are few strategies that expand physicochemical and biological features of chitosan. Quaternization of chitosan significantly improves the aqueous solubility in neutral pH hence enhances the diffusion of drug moiety across biological membrane in neutral/alkaline physiological conditions. The positive charge facilitates pronounced mucoadhesiveness, antimicrobial activity, biocompatibility, and biodegradability as well as widening its biomedical applications. Numerous trimethyl, triethyl, dimethyl ethyl, and N-(2-hydroxy-3 trimethyl ammonium) propyl derivatives of quaternized chitosans have been discussed in Table 5 as potential carriers for the transportation of proteins, genes, vaccines, and chemotherapeutics at the target site [39,129].

#### 3.6.5. Quaternized Chitosan Nanocomposites

Strong interaction between bifunctional quaternized chitosan and carbon nanocomposite displayed superior mechanical properties (tensile strength), improved ionic absorption, enhanced antimicrobial action owing to their enormous surface-to-volume ratio (surface area), smaller size, and higher dispersion in given media. Abdel-Aziz et al. (2020) have developed a novel antituberculosis delivery system composed of N,N,N-trimethyl chloride (TMC)/Ag nanocomposite synthesized through a one pot green route. Synthesized nanocomposite (11–17 nm) system has exhibited promising antimycobacterial action (MIC 1.95 μg/mL). The observed antitumor activity displayed less toxicity (IC_50_ 357.2 μg/mL) for normal (WI 38) lung cells and preeminent growth inhibition for A549 cancerous cells (IC_50_ 12.3 μg/mL) [130]. Luo et al. (2015) initially synthesized nanocomposites from chitosan/montmorillonite resin and quaternized modifier 2,3-epoxypropyltrimethyl ammonium chloride chitosan/montmorillonite resin. The developed quaternized chitosan-containing nanocomposites were small, spherical, smooth, dense, and exhibiting good dispersibility in water. The adsorption study performed on methyl orange revealed that quaternized chitosan/montmorillonite was strongly adsorbed compared to without montmorillonite, and thus can be a prospective material for column packing and wastewater treatment [131]. In this series, mechanical and ionic conductive properties of QCH functionalized carbon nanotube membrane matrix were evaluated. Improved dispersion of carbon nanotube promoted the load transfer and assisted hydroxide ion exchange through the membrane matrix. Reduced ionic conductivity and modified tensile strength indicated the potential application in preparation of anionic exchange membrane fuel cells [132]. Similarly, Gong et al. (2019) developed a layered double hydroxide ion conductor composed of QCH/PVA and carbon nanotubes. The system exhibited 1.57-fold enhanced tensile strength, 47 mS/cm^2^ ionic conductivity at 80 °C, and displayed a good reinforcing property [133].

QCh nanoparticles are highly acclaimed for designing oral drug delivery systems by virtue of significant drug diffusion and improved penetration across the epithelial barrier. Several ‘bottom up’ methods for nanoparticles development are widely emphasized, including emulsion droplet coalescence, ionic gelation, the reverse micelle method, self-assembly, chemical alteration, coacervation, and precipitation. Methods such as milling, ultrasonication, and high-pressure homogenization listed as ‘top down’ are also employed for nanoparticles synthesis [140]. Omar et al. (2021) developed novel oral drug nanocarriers composed of quaternized aminated chitosan and curcumin to enable the slow release of curcumin at the site of colon.

The ionic gelation method was opted for to formulate nanoparticles (Q-AmCs NPs) employing different concentration of crosslinker ‘sodium triphosphate’. Formulated Q-AmCs NPs were comparatively nanoscaled (~162 nm) with higher entrapment efficiency (~94.4%) and maximum potential of +48.3 mV than aminated chitosan (~231 nm, 75%, +32.8 mV). The in vitro drug release study in various physiological media (simulate gastric fluid, pH = 1.2; and simulated colon fluid, pH = 7.4) exhibited controlled cumulative drug release in alkaline pH (74%). The NPs depicted acceptable biocompatibility and biodegradability that suggested potential use for cancer management [141]. Improved solubility of quaternized chitosan in variable pH ranges encourages versatile biomedical usage [142]. Advanced applications of quaternized chitosan NPs are reported in vaccine development as well. The occurrence of cationic charge on quaternary ammonium groups is independent of any pH of surrounding media. Moreover, the positive charge facilitates advanced bioadhesion and drug delivery across mucosal membrane via strong electrostatic interaction with negatively charged endothelial regions [143].

#### 3.6.6. Vaccine Adjuvants

Vaccine adjuvants are essential components that modify vaccine potency through encouraging cell-mediated or humoral immune responses via vaccine antigens. An ideal adjuvant should have the efficacy to solubilize antigens, facilitate transportation across mucosal barrier and potential for encouraging systemic and mucosal immunity. Chitosan and chitosan derivatives are fascinating candidates for vaccine adjuvants by virtue of their remarkable physicochemical (solubility, stability, biodegradability) and biological values (cytocompatibility, non-toxicity, antimicrobial). Reports cited that quaternized chitosan have greater potential to induce antigen-presenting cells (APCs), encourage cytokine stimulation and produce preferred humoral, cellular, and mucosal immune responses [144].

A lot of chemical structure modifications have been performed with the primary amine group of chitosan to enable it to have high aqueous solubility without altering exclusive biological properties. QCh decorated with N, O-carboxymethyl and N-2-hydroxypropyl trimethyl functional moieties are frequently preferred for the synthesis of vaccine adjuvants. During vaccine preparation, the pH of QCh ranges from 4.3 to 5.5, which facilitates protonation of chitosan and makes natural electrostatic interaction with negatively charged encapsulated antigen [145]. QCh and its derivatives are utilized as potential mucosal vaccine adjuvants owing to their biological features including the formation of natural electrostatic interaction between cationic chitosan and anionic sialic acid in mucus that assists improved mucosal absorption of antigen. TMC possesses all these virtues along with higher aqueous solubility, greater mucoadhesiveness and more stability in diverse ionic conditions than chitosan; hence, it is utilized broadly in vaccine development. Moreover, TMC finds vital role in vaccine development because of its similar functional performance as that of synthetic adjuvants including alum, cyclic guanosine monophosphate and Freund’s adjuvant, etc. [146]. Moreover, QCh has a better capacity to open tight mucosa junctions than chitosan, which facilitate permeability of antigens within the mucosal cells [147]. Biocompatible, non-reactogenic, economical and biodegradable QCh are also reported for their immunomodulation ability [148].

**Figure 10:**
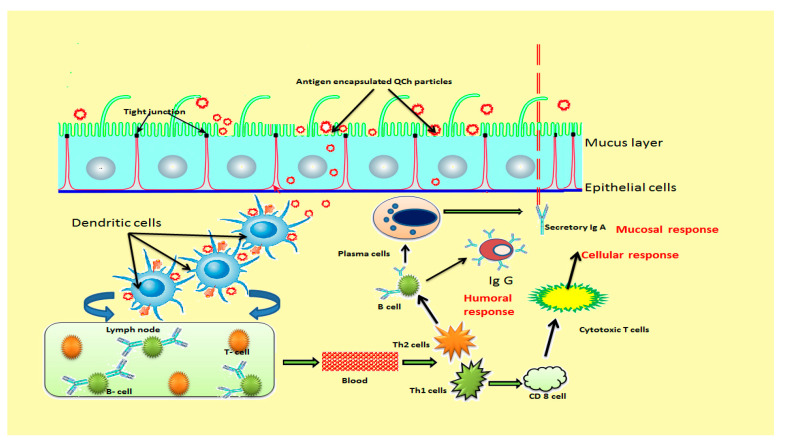
Schematic illustration of immunity development after administration of mucoadhesive QCh-based vaccine adjuvant.

Recently, many novel vaccine adjuvants have been engineered for the modification of adaptive immune responses to deliver antigen in the body that enhance the potency and maintain immunity for a long duration. Alum has been the sole vaccine adjuvant approved for human use but limits significant stimulation of cell-mediated immune responses. In this context, Tao et al. (2017) evaluated immune-modifying features of N-(2-hydroxy) propyl-3-trimethylammonium chitosan chloride (HTCC) as a vaccine adjuvant for HEV (hepatitis E virus). HTCC showed strong immune-modifying effects when co-administered with HEV recombinant polypeptide inoculum via intramuscular way. The outcomes revealed enhanced serum HE-recognized IgG antibodies, amplified CD4+, CD8^-^ T lymphocytes, proliferation of splenocytes, and growths of IFN-γ-secreting T helper cells in peripheral blood after vaccination [149]. Hence, HTCC adjuvant has the potential to induce significant antibody responses via stimulating Th-1 and Th-2 cells, cytotoxic T cells and macrophages that initiate typical mechanisms of the host defense process [150]. Th-1 cells are associated with protection against intracellular pathogens and encourage delayed-type hypersensitivity reactions, whereas Th-2 cells encounter extracellular pathogens and mediate humoral responses. Figure 10 illustrates the development of immunity after the administration of mucoadhesive QCH-based vaccine adjuvant [151].

Wu et al. (2012) extended the study and demonstrated safe usage of HTCC supported hydrogel for efficient nasal immunization. A novel thermosensitive hydrogel was developed utilizing α, β-glycerophosphate and different quaternized degree having HTCC (QD 41%, 60%, 79%, 99%). The developed adjuvant was linked with Zaire Ebola virus glycoprotein antigen and evaluated for intranasal vaccine delivery efficacy. HTCC with moderate quaternization (60% and 79%) induced high immune-modulating effects and extensively released IgG-G1-G2a antibodies in serum and IgA in lungs, probably due to prolonged resistance due to the thermosensitive nature of hydrogel. Furthermore, HTCC mediated the higher release of IFN-γ and interleukins (IL-2, IL-10, IL-4), and Th1 and Th2 cells displayed enhanced cellular immune responses [151,152].

Wang et al. (2016) developed quaternized chitosan-based microgel adjuvant for H_5_N_1_ spilt vaccine. Microgels were prepared through the premix membrane emulsification method without the addition of a chemical crosslinker. The formed polycationic QCh microgels were smaller (807 nm), pH-sensitive, biocompatible, and capable of enhancing both cellular/humoral immunity. Microgels containing QCh with a moderate degree of quaternization (41% and 60%) exhibited favorable immune responses at lower doses when investigated against bone-marrow-derived dendritic cells [153]. Traditional immunization techniques including subcutaneous and intramuscular administration can provoke systemic immunoresponses. These approaches are unable to evoke resilient local immunity, specifically in the mucosal region, the first line of defense against pathogens [154].

The nasopharynx lymphoid tissues cannot support strong mucosal immunity or immune responses. While antigen or foreign particles enter on the nasal mucosal surface, antigen presenting cells on epithelial cells identify and present them to macrophages or lymphocytes that cause their differentiation, activation, and proliferation. Non-adherent antigens are not entertained by the tightly bound epithelial cells, so they produce poor immunogenicity [155]. To overcome the above downside, Zhang et al. (2018) developed curdlan sulphate complexed O-HTCC nanoparticles adjuvant to improve immunogenicity of antigen ovalbumin (Ova) through intranasal vaccination. Soluble curdlan sulphate is an extracellular polysaccharide that particularly identifies the dectin1 receptor on immune cell surfaces. Reports cited that curdlan sulphate elicited satisfactory cellular or humoral responses. However, being anionic in nature, it is hardly adsorbed over nose epithelial cells that limit its application in mucosal immunotherapy. Hence, polycationic, immunogenic O-HTCC was chosen to make complex with negatively charged curdlan sulphate (CS) followed by entrapment of antigen ovalbumin (Ova). An efficient Ova-loaded vaccine adjuvant (Ova/CS/O-HTCC) was developed under mild conditions. When administered through nasal mucosa dripping in BALB/c mice (Bagg albino laboratory bred strain), adjuvant Ova/CS/O-HTCC activated APCs and enhanced both cellular and humoral activity of Ova. The adjuvant also ameliorated Th-2 cells responses and reduced allergic reactions [156].

Nevagi et al. (2018) developed an efficient self-adjuvating TMC-peptide vaccine based on ionic interaction between cationic TMC and anionic alpha-poly- (l-glutamic acid) peptide antigen. The designed system induced higher systemic and mucosal antibodies compared to the reference cholera toxin B, a mucosal adjuvant conjugated with TMC. A self-adjuvant evident reduction in bacterial burden in nasal secretions of immunized mice was thus found to be effective for group A *Streptococcus* pathogen [157].

## 4. Miscellaneous

### 4.1. Orthopedic Surgery

Biomaterial-related infections are serious glitches in orthopedic surgery. The application of antibiotics either locally or given systemically has proven valuable in clinical research. Bone cements are applied to deliver antibiotics locally in high doses without triggering systemic toxicity. However, at a high level, these antibiotics may reduce the osteoblasts cell viability, proliferation, and alkaline phosphatase action that hinders physiological functions of bone marrow mesenchymal cells, resulting in compromised bone healing process. To overcome the above issue, quaternized chitosan derivative ‘hydroxy-propyl-trimethyl ammonium chloride chitosan (HACC)’ loaded on polymethylmethacrylate (PMMA) bone cement was investigated to resolve the formed biofilm on the surface of bone cement. HACC-functionalized PMMA bone cement exhibited better antibacterial action compared to control against versatile clinically isolated microbes including *S. epidermidis 389*, methicillin-resistant *S. epidermidis* and methicillin-resistant *S. aureus* [158]. The developed system proved to have the potential to combat implant infections related to osteomyelitis. Moreover, the findings demonstrated better stem cell proliferation; osteogenesis and cell differentiation on the surface of HACC-loaded bone cement compared to antibiotic gentamycin-loaded bone cement [159]. Therefore, quaternized chitosan-based derivatives HACC finds biomedical roles in osteogenic activity, arthroplasty, and gene expression osteogenesis [160].

Furthermore, HACC with 6%, 18% and 44% degrees of quaternization coated on the titanium surface have been utilized for the evaluation of biofilm preventing efficiency. Titanium-based bioactive agents are widely used orthopedic implants due to their biocompatibility, mechanical strength, corrosion resistance and light weight. However, these implants provide a favorable environment for bacterial colonization followed by biofilm formation. Thus, prolonged application was compromised with defective osteoproliferation, poor osteogenesis and serious microbial infections. HACC-coated titanium implants would overcome the above issues with an enhanced anti-infective effect at the same time. HACCs (18% and 44% degree of quaternization) were biocompatible with osteogenic cells and significantly eradicated the developed biofilm around orthopaedic implants at very low MIC values [161].

Yang et al. (2016) extended biomedical applications of HACC-modified titania nanotubes to improve the in vivo anti-infection potential. Orthopaedic intramedullary nails are widely used for the treatment of open and closed ‘tibial and femoral’ fractures. However, there may be postoperative infections due to implanted intramedullary nails [162]. A high dose of antibiotics is recommended that causes systemic toxicity and osteomyelitis, and delays bone cells proliferation. Hence, implants with an advanced anti-infective property are required to fix the high rate of infections. This innovative research detailed formation of various titania nanotube (NT) loaded with HACC (NT-H) and chitosan (NT-C) and evaluated their comparative cytocompatibility, antimicrobial efficiency and osteoblastic activity against human bone marrow mesenchymal stem cells. The developed NT-H depicted impressive osteogenic action compared to other NT and NT-C nanotubes when assessed on a rat model having femoral medullary cavity implantation and methicillin-resistant *S. aureus* infection. Superior antibacterial effect, cytocompatibility, and profound osteogenesis focused the clinical application of NT-H to manage orthopedic surgical implant-related infections.

Kadama et al. (2021) studied the antibacterial effect of HACC-coated 3D-printed titanium cage during the treatment of improper intervertebral disc space in a rat model. Ti-HACC and non-coated Ti with bioluminescent *S. aureus* were inserted in the caudal discs of model rat. From the in vivo imaging system (IVIVS), the post-operative infection-related changes such as bone destruction and the movement of the implanted cage were assessed on the first, third, and fifth day through micro-computer tomography. The outcomes defined the vital role of Ti-HACC for effective wound healing, improved tartrate-resistant acid phosphate-positive osteoclasts, better antibacterial action against *S. aureus* and preservation of structural damage in caudal disc in rat [163].

### 4.2. Dental Care and Cosmetics

Chitosan derivative N-[(2-hydroxy-3-trimethylammonium) propyl chitosan chloride, HTCC has better solubility in neutral and alkaline solutions. HTCC has been utilized for the preparation of dental care products owing to its enhanced antimicrobial action [164]. Quaternized chitosan and its substituents have promising action in reducing dental plaque associated with various pathogens. *Porphyromonas gingivalis*, *Actinobacillus actinomycetemcomitans*, *Streptococcus mutans* and *Prevotella intermedia* cause plaques and periodontal diseases. Endodontic cements based on QCh derivatives reduce inflammation and facilitate bone regeneration [165]. Literature reports promising antibacterial action of HTCC against *Enterococcus faecalis* that is associated with endodontic infection. *E. faecalis,* a Gram-positive bacteria, has been isolated from persistent endodontic infection that may cause severe root canal disorder. This pathogen may exist in non-culturable conditions and maintain viability through the year without requiring nutrients, thus damaging dentinal tubules and develop biofilms. Once biofilm is formed, *E. faecalis* becomes more virulent, can protect itself either from adverse condition or high doses of antibiotics [166].

Patel et al. (2020) explored the antibacterial efficiency of chitosan and HTCC against *E. faecalis* pathogen in the state of planktonic and biofilm. A colony-forming-unit study was performed to define the status of three strains of *E. faecalis* (ATCC 29212, P25RC, P52Sa) after treating with serially diluted (20–2500 μg/mL) chitosan and HTCC. Both chitosan (70 μg/mL) and HTCC (140 μg/mL) exhibited significantly higher antibacterial efficacy in double-distilled water as compared to phosphate-buffered saline. The presence of charged ions (Na^+^, K^+^, Cl^−^ and H_2_PO_4_^−^) in phosphate-buffered saline might disrupt the involved electrostatic interaction that results in less antibacterial action [167]. Moreover, the biocompatibility of natural biopolymer chitosan and its derivative HTCC for the purpose of sealing/obturation of the root canal is far better, compared to other traditionally employed toxicity elicited substances such as zinc oxide, glass ionomers, resins, calcium hydroxide, etc. [168].

Thus, the biocompatibility and characteristic antibacterial efficacy of both chitosan and HTCC focus biomedical application in root canal therapy [169]. Lee et al. (2018) examined the antifungal activity of quaternized chitosan ‘2-[(acryloyloxy) ethyl] trimethylammonium’ as tissue conditioner in the pathological condition ‘denture stomatitis’. *Candida albicans* is a main etiological cause for denture stomatitis that affects mucosa underneath diseased teeth. QCh-based tissue conditioner of size 1 mm thickness and 10 cm diameter was prepared and co cultured with *C. albicans*. Significantly diminished fungal colonies were observed with less than 5% QCh concentration compared to the resin denture base. Moreover, this amount did not affect the viability of gingival epithelium cells [170]. Table 6 collects novel research reports on QCh-based systems that have been explored for dental and cosmetic applications.

Quaternized chitosan derivatives are highly utilized for the preparation of cosmetics including skin creams, shampoo, sprays, and dental remedies. Being natural with exclusive biological features, these biopolymers find an attractive domain in cosmeceuticals. Water-soluble quaternized chitosan (73% degree of quaternization) has been grafted with cyclodextrin (QCD-g-CS) to improve its antibacterial effect against several microorganisms, namely *C. albicans*, *S. mutans*, and *S. oralis* [171].

Sakulwech et al. (2018) extended the study by uploading the negatively charged hydrophilic guest (hyaluronic acid) on the QCD-g-CS to improve penetration enhancement. Hyaluronic acid is generally applied in skin remedies owing to improved skin elasticity, an antiwrinkle effect and the retention of moisture content. Furthermore, it is popularly used for treating osteoarthritis and manifesting wound healing. The high molecular weight of hyaluronic acid limits its benefit in cosmetics. Amalgamation of quaternized chitosan and cyclodextrin facilitated enhanced solubility, absorption, and bioavailability to the dermal cells. The developed nanoparticles enhanced the penetration of hyaluronic acid in deeper tissues and provided a moisturizing effect. The developed nanoparticles were safe when investigated against skin fibroblast cells [174].

Khalaji et al. (2020) developed biocompatible and antibacterial scaffolds with a blend of synthetic PVA and natural water-soluble HTCC. Fabricated scaffolds exhibited sufficient mechanical strength that was appropriate for skin regeneration during dermal damage. Moreover, the embedded collagen provided additional biological properties, such as extracellular matrix simulation, better cell attachment and proliferation. Hence, fabricated scaffolds can be medically utilized as skin care products [175]. Chen et al. (2017) developed nanocomposite containing quaternized carboxylated chitosan and organic montmorillonite through solution-induced intercalation. A cosmetic cream against skin aging was prepared that exhibited better moisture adsorption, efficient UV protection and moisture retention behaviour without dermal irritation owing to the presence of quaternized carboxylated chitosan [22].

## 5. Future Prospective

Quaternized chitosan (QCH) derivatives have been extensively utilized for biomedical applications (tissue engineering, drug/gene delivery, antimicrobial, antiviral and immunoadjuvant) owing to their bio and cytocompatibility attributes. With the advancement of nanotechnology, QCH together with nanocomposites (carbon nanotubes and nanofillers) provide superior alternatives to the new arena of drug delivery and regenerative medicines. The reports and findings detailed that QCH derivatives are increasingly focused for the formulation of vaccine adjuvants, chemotherapeutic agents, ecofriendly textiles, and purified membrane matrix. Improved physicochemical properties including improved solubility, mucoadhesiveness, absorption and bioavailability pave QCH derivatives for the synthesis of less invasive and more effective formulations for the management of diverse disorders.

## 6. Conclusions

Multifunctional QCh derivatives have attracted high attention in the last two decades by their inherent virtues that have broadened biomedical applications. Reports cite extensive research on QCh-based drug carriers, vaccine adjuvant, anticancerous nanoparticles, antibacterial hydrogel, tissue scaffolds, and purification membranes that explore their exclusive physiochemical and biological actions. Furthermore, QCh derivative polymers exhibit high stability, biodegradability and biocompatibility that are essential benchmark for successful therapeutic remedies. However, the synthesis of QCh derivatives including pyridinium and phosphonium salts should be properly channelized at an industrial level to expose their superior biomedical applications on a large scale.

## Figures and Tables

**Table 1:** Effect of parameters influencing the biological responses [20,21].

Parameters	Responses
Degree of quaternization (<65%)	Increased cytotoxicity
	Increased mucoadhesiveness
	Decreased anticoagulation effect
Degree of quaternization (≥20%)	Increased antimicrobial action in pH = 7.2
	No effect on antimicrobial action in acidic pH
Degree of substitution (<1%)	Increased antioxidant property
Degree of substitution (<25%)	Increased antithrombin action and acid-binding capacity
Degree of substitution (>1%)	Decreased moisture absorption and retention ability
High concentration	Increased particle size, aggregation, zeta potential, cytotoxicity
	Decreased knockdown efficiency and poor transfection efficacy

**Table 2:** Newly introduced quaternized chitosan derivatives and their biomedical application.

Quaternized Chitosan Derivatives	Biomedical Applications	References
N,N,N-trimethyl-O-(2-hydroxy-3-trimethylammonium propyl) chitosan (TMHTMAPC)	Antimicrobial against *S. aureus* and *E. coli* in both acidic and alkaline pH.	[37,54]
Water soluble N-piperazine chitosan	Pharmaceutical application	[55]
Water soluble N-betainate chitosan	Antibacterial for *S. aureus* and *E. coli* Effect of antibacterial efficacy decreased with increase in amino group substitution.	[48,56]
N,N-dimethyl-O-quaternary ammonium chitosan	Cosmeceuticals (moisturizer), antibacterial	[57]
O-acetyl-chitosan-N-2-hydroxypropyl trimethyl ammonium chloride	Antibacterial (*S. aureus* and *E. coli*), Food preservative	[58]
Amphiphilic 5-(bromo pentyl) trimethyl ammonium conjugated with dodecyl aldehyde chitosan (BPTADDACH)	Efficient drug carrier for hydrophobic candidates	[59]
Diethylaminoethyl-2-N-carboxymethyl-6-O Chitosan (DEAE–CMC) containing vitamin B12 microspheres	Blood compatible drug delivery system	[60]
N,N,N-(diethyl aryl) chitosan chloride	Antifungal/fungicide for crop threatening *Botrytis cinerea.*	[61]
Quaternized N-(aryl) chitosan	Modified antimicrobial action against *E. coli*, *P. aeruginosa*, *S. aureus* and *A. niger* due to presence of phenol hydroxyl group	[62]
Quaternized carboxymethyl chitosan containing guanidine groups (QGCMC)	Food preservative (enhanced shelf life of strawberries), strong bactericidal action in all ranges of pH (acidic, alkaline and neutral)	[63]
O-quaternary ammonium N-acyl thiourea chitosan (OQCATUCS)	*Synergistic antimicrobial action for S. aureus*, *E. coli*, *A. niger*, *P. aeruginosa* and *B. subtilis*	[64]
N,O-[N,N-diethylaminomethyl (diethyl dimethylene ammonium) n] methyl chitosan	Intraocular drug delivery (Enhanced permeability of dexamethasone)	[51,65]
Diethylaminoethyl-2-N-carboxymethyl-6-O Chitosan (DEAE–CMC) containing vitamin B12 microspheres	Blood compatible drug delivery system	[60,66]

**Table 3:** Quaternized chitosan-embedded nanofibers in diverse biomedical arena.

Components	Purpose	Research Outcomes	References
HTCC and PVA	Retention of non-enveloped virus on the highly charged HTCC/PVA nanofibers.	Nano-scaled HTCC/PVA nanofibers (100–200 nm) were developed, having the potential to adsorb mammalian virus porcine parvovirus (95%). The developed system followed Freundlich isotherm and showed fast adsorption kinetics (pseudo first order), which suggested the formation of efficient filter material for the purification of water	[110]
Doxorubicin, poly (L-lactide-coD, L-lactide) and QCh	Doxorubicin embedded poly (L-lactide-coD, L-lactide) mats were modified with QCh to enhance anti-proliferative activity.	Developed mats were evaluated against human breast carcinoma cell lines (MCF-7) and exhibited reduced cell viability and amplified antiproliferative activity. Fluorescent microscopy revealed that the presence of QCh induced apoptosis, which was the primary mechanism of MCF-7 cell death.	[111]
2,3-Epoxy-propyl trimethyl ammonium chloride	QCh fibres were designed using 2,3-epoxypropyl trimethyl. Ammonium chloride following ring open reaction to modify antibacterial and liquid absorption capacity.	Outcomes revealed excellent water retention capacity, modified swelling index and mechanical strength compared to bare chitosan. Superior antibacterial efficacy against *S. aureus* and lower cytotoxicity suggested its vital role in fabricating wound dressing materials.	[112]
Poly (lactic acid), QCh	Stereo complex crystallite (SC) membrane containing poly (lactic acid) QCh were employed to design disinfectant wound dressing material.	The enhanced thermal and mechanical properties of developed SC membrane owing to restricted mobility of lactide chains. They have better wound healing capacity (100% in 15 days). This biomass-based membrane was multifunctional as it has antioxidant, antibacterial and wound healing efficacy.	[113]
Silica coated poly (vinylidene) fluoride and QCh	High-performance anion exchange silica-coated (vinylidene) fluoride along with QCh nanofibrous membrane were designed.	The surface of silica-coated poly (vinylidene) fluoride was grafted with quaternized chitosan to pursue dual action, i.e., ion exchange and strong reinforcement substrate. QCh-impregnated nanofibers showed superb mechanical strength (11.9Mpa). Adorned positive charges created channel-like ion transport channels that could efficiently serve as anion exchange membrane.	[114]

**Table 4:** Biomedical utility of QCh-derived hydrogels.

Objective	Components	Research Highlights	References
Multifunctional QCh-based polyacrylamide hydrogel was developed that contained hemostatic and skin adhesive properties	QCh, Matrigel-polyacrylamide	The developed hybrid hydrogel had a three-dimensional microporous integrity and exhibited high mechanical strength and good adhesiveness with low toxicity. The outcomes from the histology study demonstrated improvement in wound healing, collagen deposition, and stimulation of skin adnexal regeneration. The developed QCh-based antibacterial hydrogel demonstrated promising potential for designing wound dressing materials.	[118]
Dual crosslinked QCh-clindamycin loaded hydrogel was prepared to manage methicillin-resistant *S. aureus* (MRSA) bacteria	QCh, clindamycin	The developed nanocomposite-embedded hydrogel withstood sufficient mechanical and injectable efficiencies. The system responded on variable pH that enabled maximum interaction with MRSA bacteria (90% killed) in acidic conditions and overcame the antibiotic resistance challenge.	[119]
A novel wound dressing-based injectable hydrogel was designed employing QCh and PLEL (PLEL-nBG-QCS-C) hydrogel to promote angiogenesis.	QCh and PLEL [Poly (D, L-lactide)-poly (ethylene glycol)-poly (D,L-lactide)] and bioactive glass	PLEL hydrogels preloaded with bioactive glass (CaO-SiO_2_-P_2_O_5_) could efficiently seal the broken skin and increase the cure rate of wounds. Additionally, they were thermosensitive, tissue adhesive, and antibacterial.	[120]
QCh-based timolol maleate thermosensitive hydrogel was prepared for improved ophthalmic disorders.	Timolol maleate, Sodium hydrogen carbonate, QCh	The developed transparent thermosensitive hydrogel presented desirable porosity, swelling index, and biodegradability. The addition of sodium hydrogen carbonate enabled enhanced thermosensitivity to the system. In vitro drug release revealed the initial burst release in early hours followed by controlled release of timolol maleate for a week. This supported the potential use of the developed hydrogel for glaucoma management.	[121]
Dopamine-gelatin-crosslinked QCh injectable hydrogel was prepared to localize delivery for the combat of Parkinson and associated inflammation as well.	Dopamine, QCh, Metronidazole, gelatin	The formulated injectable hydrogel exhibited sufficient rheological parameters. The cytocompatibility of hydrogel revealed the cell viability and proliferation of L929 fibroblast cells. In vitro study exposed localized release of both dopamine and metronidazole.	[122]
QCh-based pH-sensitive veterinary hydrogel vaccine for improved cellular and humoral responses.	QCh, Montanide^TM^ ISA206 and glycerophosphate	The developed hydrogel was biocompatible, safe, and had efficiencies to adsorb inactivated porcine reproductive and respiratory syndrome virus. Moreover, the system ruled out the downsides of mineral oil side effects and encouraged immunogenicity.	[123]
Development of NQC-loaded thermostable and multifunctional hydrogel	N-quaternized chitosan (NQC), poly vinyl alcohol, glutaraldehyde	Different hydrogels on varying concentration of NQC and PVA were designed to modify metal ion uptake, swelling capacity, compatibility, and antibacterial efficacy.	[124]

**Table 5:** QCh-based nanoparticles and their biomedical applications.

Objective	Components	Research Highlights	References
Ketoconazole was entrapped in QCh NPs for superior antifungal activity	Ketoconazole, QCh, sodium triphosphate	Nanoscaled KCZ-QCSNPs displayed superb entrapment efficiency (~90%). Performed tube dilution method revealed preeminent antimicrobial activity.	[134]
QCh derivative ‘HTCC’ NPs were embedded in various fabric materials to evaluate antimicrobial efficacy.	HTCC, cotton fabric, polyester, polyacrylic acid	The developed HTCC nanoparticles embedded in cotton fabric exhibited superior antimicrobial action against *Fusarium oxysporum* and *Bacillus subtilis* compared to polyester and mixture of cotton.	[135]
Anthrax vaccine adjuvant containing Fucoidan-HTCC nanoparticles were developed to improve rapid induction of immunity	Sulphated polysaccharide (Fucoidan, FUC) and HTCC	An active complexation between opposite-charged FUC and HTCC was conducted through varying their mass ratio. MTT assay on L929 or JAWS dendritic cells evaluated low cytotoxicity, improved cellular internalization and high cell viability. Combination of FUC-HTCCNPs and anthrax vaccine adsorbed (AVA) significantly improved magnitude of cellular/humoral immunity and mice survival rate compared to administration of AVA alone.	[136]
Nanoparticles containing N-2-HTCC and N,O-CMC encapsulated vaccine antigens (IBV/H120) were developed for significant increments in lymphocytes, interleukins, and interferon in chicken	N-2-HTCC, N,O-carboxy methyl chitosan (CMC), infectious bronchitis virus (IBV)/H120 and Newcastle disease virus (NDV)	The developed nanoparticles, i.e., N-2-HTCC-CMC/NDV/IBV, predicted great stability and low cytotoxicity on storing at 37 °C for 3 weeks. In vivo assay on chicken revealed sustained release of both NDV and IBV with enhanced release of IgG and IgA that facilitated the proliferation of immune modifiers in chicken body. The developed QCh-based NPs showed the potential to combat respiratory diseases in chicken.	[137]
Ecofriendly QCh derivative HTCC nanoparticles were designed to increase the durability and microbial resistance of *Antheraea pernyi* silk fabric.	HTCC and 1,2,3,4 butane tetracarboxylic acid, sodium hypophosphite	The conventional dip-and-dry-cure method was applied to evaluate silk fabric durability (*A. pernyi*). Wrinkle resistance, microbial resistance (against *S. aureus* and *E. coli*) and shrinkage resistance were observed even after washing *A. pernyi* silk fabric more than 50 times.	[138]
5-flurouracil (5-FU) embedded HTCC NPs developed for improved entrapment efficiency and in vitro release	5-FU, HTCC, sodium tripoly-phosphate (TPP)	5-FU/HTCC NPs were prepared through ionic gelation method via electrostatic interaction between positive-charged HTCC and negative-charged TPP. Encapsulated 5-FU exhibited controlled release profile in pH 7.4 buffer.	[139]

**Table 6:** Research highlights few dental/cosmetic uses of QCh derivatives.

QCh-Based System	Biomedical Application	References
Conjugates of chitosan and HTCC	Antibacterial activity against oral pathogen	[172]
Thermosensitive CS-HTCC/GP-Chlorhexidine hydrogel	Local drug delivery for periodontal treatment	[135]
CS-HTCC/GP thermosensitive hydrogel	Antibacterial efficient activator for periodontal pathogens such as *Porphyromonas gingivalis*, *Actinobacillus actinomycetemcomitans* and *Prevotella intermedia*.	[173]
Conjugate of Quaternized carboxymethyl chitosan (QCMC) and calcium hydroxide	QCMC strongly induced reparative dentine formation and showed a better ability in dentin inducing compared with calcium hydroxide.	[162]

## Data Availability

Not applicable.
